# A topical BRAF inhibitor (LUT-014) for treatment of radiodermatitis among women with breast cancer

**DOI:** 10.1016/j.jdin.2023.11.009

**Published:** 2023-12-25

**Authors:** Sanford Katz, Doug Ciuba, Antoni Ribas, Noa Shelach, Galit Zelinger, Briana Barrow, Benjamin W. Corn

**Affiliations:** a Division of Radiotherapy, Willis-Knighton Cancer Center, Shreveport, Louisiana; b Radiation Oncology of Columbus, Columbus, Georgia; c Department of Medical Oncology, University of California Los Angeles (UCAL) and Jonsson Comprehensive Cancer Center, Los Angeles, California; d Lutris-Pharma, Tel Aviv, Israel; e Department of Oncology, Hebrew University Faculty of Medicine, Jerusalem, Israel

**Keywords:** BRAF inhibition, breast cancer, DLQI, hypofractionation, LUT014, radiation dermatitis

## Abstract

**Background:**

Modern radiotherapy is associated with dermatitis (RD) in approximately one-third of patients treated for breast cancer. There is currently no standard for treating RD.

**Objective:**

The objective of this study was to determine whether LUT014, a topical BRAF inhibitor which paradoxically activates mitogen-activated protein kinase, can safely improve RD.

**Methods:**

A phase I/II study was designed to first follow a small cohort of women with grade 2 RD regarding toxicity and response. Then, 20 patients were randomized to compare LUT014 to “vehicle” relative to safety and response (measured with common terminology criteria for adverse events, Dermatology Life Quality Index).

**Results:**

No substantial toxicity (eg, 0 serious adverse event) was associated with LUT014. All 8 women receiving LUT014 achieved treatment success (5-point Dermatology Life Quality Index reduction at day 14) compared to 73% (8/11) on the placebo arm (*P* = .591). The time to complete recovery was shorter in the treatment arm.

**Limitations:**

The sample size was limited. Only 2 hospitals were included.

**Conclusions:**

Topical LU014 is tolerable and may be efficacious for grade 2 RD.

**Capsule Summary**

• LUT014, a BRAF inhibitor, was recently shown to mitigate the papulopustular rash associated with epidermal growth factor receptor inhibitors. We hypothesized that this topically applied gel could be used to treat radiodermatitis.
• LUT014 was well-tolerated and was associated with improvements that did not achieve statistical significance.

## Introduction

Local control benefits and modest survival improvements are associated with breast-conserving radiotherapy (RT) for women with early-stage breast cancer.^1^ Unfortunately, it is axiomatic that RT can cause morbidity. Acute radiation dermatitis (RD) continues to be a common problem, with nearly one-third of patients developing moist desquamation during treatment with conventionally fractionated RT.^2^ While hypofractionation is associated with less acute radiodermatitis, patients must still cope with skin complications.^3^

Several investigators proposed creative interventions to manage RD. To date, none of these approaches have achieved regulatory approval.^4^^,^^5^ Consequently, conventional practice has been to recommend unproven commercially available products.^6^^,^^7^ Many products are costly, which results in substantial out-of-pocket expense, if not financial toxicity.^8^

Patients often fear the toxic effects of RT.^9^^,^^10^ Thus, radiation-induced dermatitis constitutes a clinically meaningful, unmet medical need. In 2020, guidelines for treating radiodermatitis were published by the Oncology Nursing Society.^11^ A subsequent editorial by Marquez and Wong,^12^ however, underscored that these Oncology Nursing Society guidelines were unable to furnish “strong recommendations.” Marquez and Wong, representing the American Society of Radiation Oncology, cautioned that strategies for radiodermatitis are predicated on expensive products that are not well-supported by evidence. Therefore, no standard of care for treating radiation dermatitis had been established. Similarly, in 2023, the Multinational Association of Supportive Care in Cancer issued guidelines for RD after convening a modified Delphi consensus process.^13^ Those investigators concluded: “a gold standard treatment for acute radiation dermatitis has yet to be established.”

Recently, Lacouture et al^14^ described a topical inhibitor of BRAF (LUT014) showing activity against papulopustular eruptions caused by epidermal growth factor receptor inhibitors. That phase I clinical trial included patients with colorectal cancer. Patients assigned to the 2 lower dosages after presenting with a grade 2 rash experienced reduced severity of skin toxicities. The topical therapy was deemed efficacious and safe. The reduction of cutaneous toxicity with the combination of epidermal growth factor receptor inhibitor and topical BRAF inhibition was attributed to a paradoxical activation of the mitogen-activated protein kinase (MAPK) pathway induced by LUT014, a novel BRAF inhibitor, thereby offsetting the suppression of this pathway brought about by the epidermal growth factor receptor inhibitor.^15^, ^16^, ^17^, ^18^

Although the mechanism of radiation dermatitis has not been fully characterized, it has been posited that RD arises from an imbalance in proliferative processes and the destruction of cells in the skin’s basal layer.^19^ An important dimension of MAPK pathway activation is the enhanced cell proliferation which ensues. This effect was assayed in Mia PaCa cells where LUT014 was observed to stimulate a dose-dependent cell proliferation.^14^ We hypothesized that LUT014 could balance the cell destruction of RD.

The current trial was designed to evaluate whether LUT014 – topically applied for 4 weeks after appearance of radiodermatitis – could improve grade 2 dermatitis of breast RT. The study also assessed symptoms of RD and adverse events.

## Methods

The present study describes a phase I/II open label trial (part 1) followed by a double-blind, placebo-controlled (part 2) clinical study with the schema presented in the Supplementary Fig 1, available via Mendeley at https://data.mendeley.com/datasets/xx9b9bkzy7/1. Part 1 was conducted at a single tertiary facility (Willis-Knighton Cancer Center, Shreveport, LA) for adult women diagnosed with breast cancer (stages TIS-T3, *N*0-2, M0). Patients were treated with either conventional fractionation or hypofractionated techniques. Patients who enrolled in the protocol had both grade 2 dermatitis (common terminology criteria for adverse events, version 5.0)^20^ and a Dermatology Life Quality Index^21^ score of >6 at screening and baseline. Of note, the protocol specified that patients complete fractionated RT *prior to* receiving the first dose of study drug. Part 2 targeted the same patient populations but was also conducted at a second site (Amos Cancer Center).

The protocol called for topical administration of 0.3 mg/g LUT014 gel or placebo on a daily basis for 28 days. To avoid toxicity from combining BRAF inhibition with RT,^22^ LUT014 was administered after the final fraction of RT was delivered. A thin layer of gel was applied to the radiodermatitis. The gel was administered on day 0 under supervision, but thereafter was applied by the subject at home. The initial plan was to recruit 15 adult women for the study; however, when consistently favorable results were observed for 8 patients, the protocol was modified to introduce a placebo control arm to rule out the likelihood of confounding from natural healing or a therapeutic benefit of vehicle. As such, this phase I/II trial comprised a nonrandomized as well as a randomized component. Of note, in the randomized portion of the study, due to arbitrary prescribing patterns of the physicians, subjects on the control arm applied the placebo gel at a median of 3 days after completion of irradiation, whereas subjects on the experimental arm applied the LUT014 gel at a median of 4.2 days after completion of RT.

In the initial portion of the study, the primary objective was to evaluate the safety and tolerability of LUT014. A secondary objective was to determine whether a therapeutic signal was evident and worthy of more rigorous testing. The protocol is available online (ClinicalTrials.Gov: NCT04261387). Human use approval was secured from an institutional review board of the participating institutions. In the randomized portion of the trial, the primary end point was the treatment success rate in the experimental arm vs the placebo arm. Success rate was defined as an improvement of at least 5 points in the Dermatology Life Quality Index score at day 14.

## Results

### Patient characteristics

Patient characteristics are portrayed in the Supplementary Table I, available via Mendeley at https://data.mendeley.com/datasets/xx9b9bkzy7/1. The distribution of factors typifies the clinical features encountered in the practice of RT. With regard to the randomized component of the trial, it is noteworthy that the group receiving LUT014 tended to have a higher body mass index than those randomized to placebo.

### Safety and tolerability of LUT014

In the open-label component of the trial there were – in total – 33 nonserious adverse events (AEs), of which 28 were unrelated to LUT014 Gel and 5 were adverse drug reactions that were reported among 3 subjects. The 5 adverse drug reactions were mild. Two of the patients reported mild skin burning which was transient. One patient reported mild breast pain, which resolved within a week, and 1 patient reported pruritis. No serious adverse events were encountered in the open-label component of the trial.

During the randomized portion of the trial, overall, there were 46 AEs reported for 15 subjects (8 subjects in the LUT014 Gel and 7 in the placebo group experienced an AE). Of the 46 AEs reported, 21 adverse events arose in the LUT014 Gel group and 25 adverse events were encountered in the placebo group. Only 5 AEs (which arose in 4 patients) were related to the LUT014 itself. It is noteworthy that no serious adverse events were observed. It can be seen from Table I that there were four AEs (in 3 subjects) which were considered related or possibly related to the study drug in the LUT014 Gel group (2 mild skin burning, 1 mild pruritus, and 1 moderate skin burning) as well as one mild AE considered related to the treatment in the placebo group (mild skin burning). There were no serious AEs observed. Of note, 1 patient dropped out before the day 7 visit due to an AE that was unrelated to the study drug.

**Table I:** Summary of adverse events for open label and randomized double-blind phase I/II trial

	Open label LUT014 gel				LUT014 gel				Placebo			
	*N* (8 subjects)	% subjects	*n* (AEs)	% of total AEs	*N* (9 subjects)	% subjects	*n* (AEs)	% of total AEs	*N* (11 subjects)	% subjects	*n* (AEs)	% oftotal AEs
AEs	8	100.0	33	100.0	8	88.9	21	45.7	7	63.6	25	54.3
SAEs	0	0	0	0	0	0	0	0	0	0	0	0
ADR	3	37.5	5	15.2	3	33.3	4	8.7	1	9.1	1	2.2
Mild AEs	8	100	31	93.9	8	88.9	17	37	7	63.6	23	50.0
Moderate AEs	2	25	2	6.1	1	11.1	4	8.7	2	18.2	2	4.3
Severe AEs	0	0	0	0	0	0	0	0	0	0	0	0
Life-threatening AEs	0	0	0	0	0	0	0	0	0	0	0	0
AE leading to study discontinuation	0	0	0	0	1	11.1	1	2.2	0	0	0	0
AE leading to death	0	0	0	0	0	0	0	0	0	0	0	0
UAEs	7	87.5	22	66.7	6	66.7	10	21.7	6	54.5	12	26.1
ADR and UAEs	1	12.5	1	3.0	0	0	0	0	0	0	0	0

*ADR*, Adverse drug reaction; *AE*, adverse event; *SAE*, serious adverse event; *UAE*, unanticipated adverse event.

### Identification of possible effect in first component of the trial

**Fig 1:**
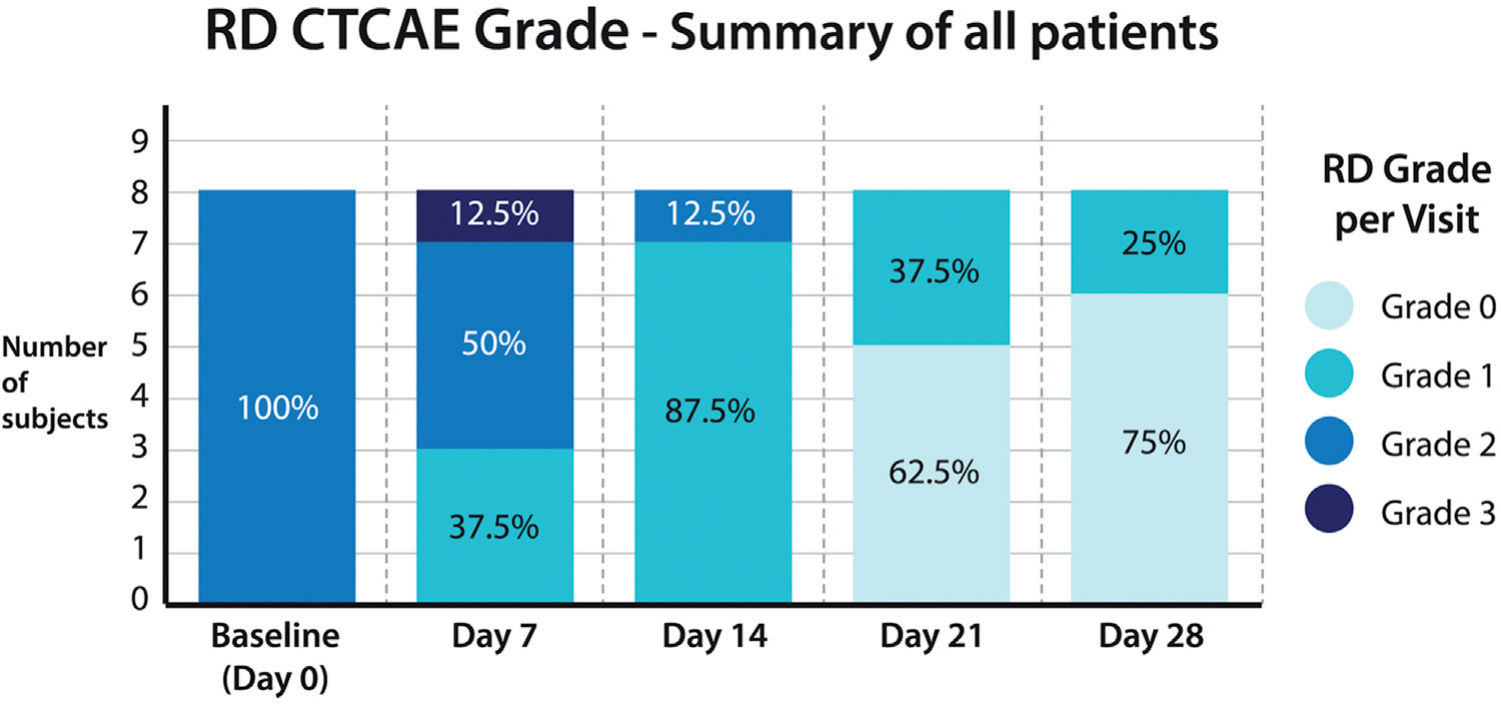
Summary of the grading of results from the first component of the trial. At baseline, all patients had grade 2 dermatitis. After 1 week, 37% improved to grade 1 and 1 patient deteriorated to grade 3. After 2 weeks of treatment, 87% improved to grade 1. By 3 weeks, 62% had complete recovery (grade 0) and 37% had grade 1 RD. At the end of the study, 75% had grade 0, while 25% had improvement to grade 1. *RD CTCAE*, Radiation dermatitis common terminology criteria for adverse events.

**Fig 2:**
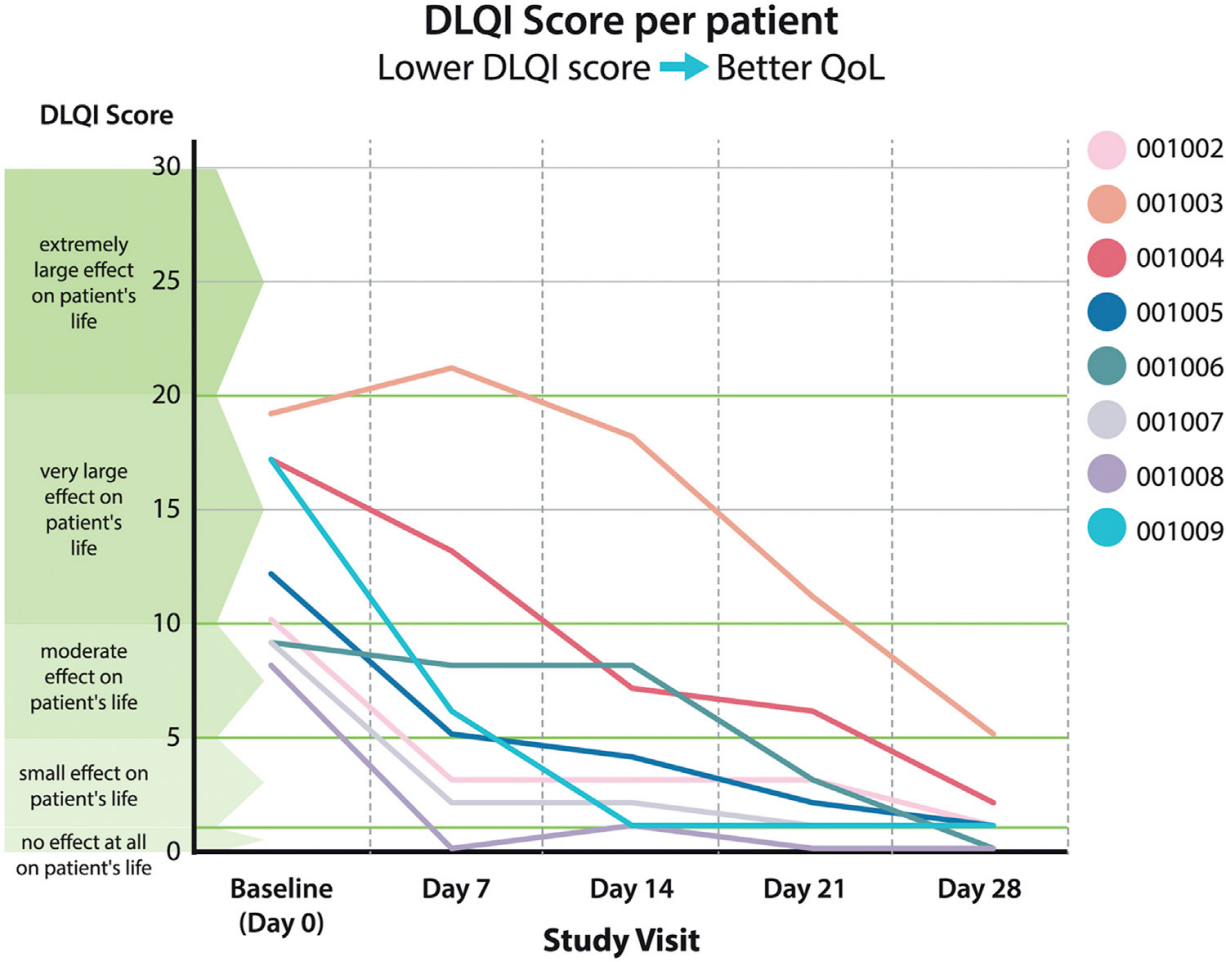
Dermatology Life Quality Index (*DLQI*) among patients treated on protocol. Of note, a DLQI score higher than 10 indicates that a patient’s life is being severely affected by their skin.

Fig 1 displays the data for all patients in the first component of the trial. By day 28, 6 of 8 patients who developed grade 2 dermatitis at baseline improved to grade 0 dermatitis (ie, complete resolution). Fig 2 shows the marked improvement of life quality reported by the patients enrolled in the first phase of the trial. Consistent improvement was observed in the quality of life of all the patients, from “very large effect on subjects’ life” (5 patients) or “moderate effect on subject’s life” (3 patients) at baseline to “small effect on subjects’ life” (2 patients) or “no effect on subjects’ life“(6 patients) at day 28.These data were deemed to be sufficiently intriguing to trigger the randomized component of this phase I/II trial.

### Randomized double-blind component of the trial: Efficacy

**Fig 3:**
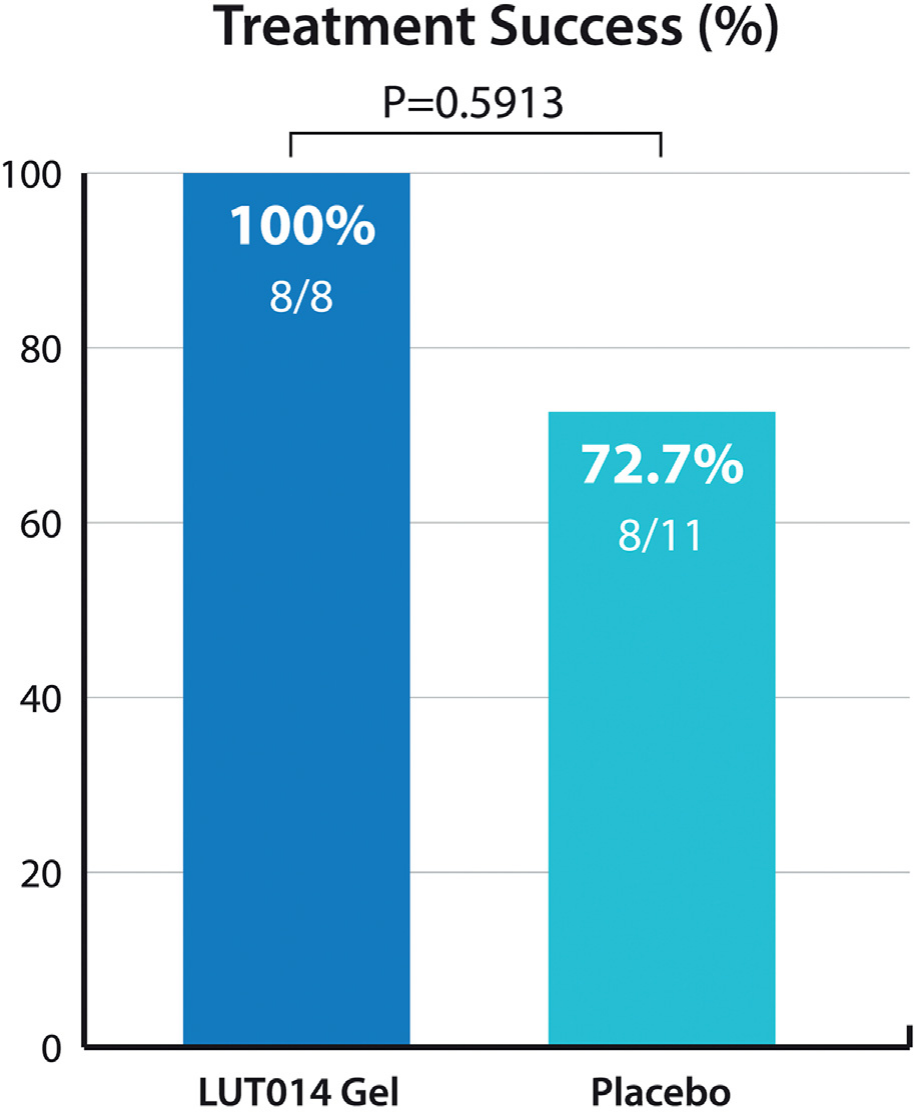
Rates of “treatment success” as defined for the randomized component of the study.

**Fig 4:**
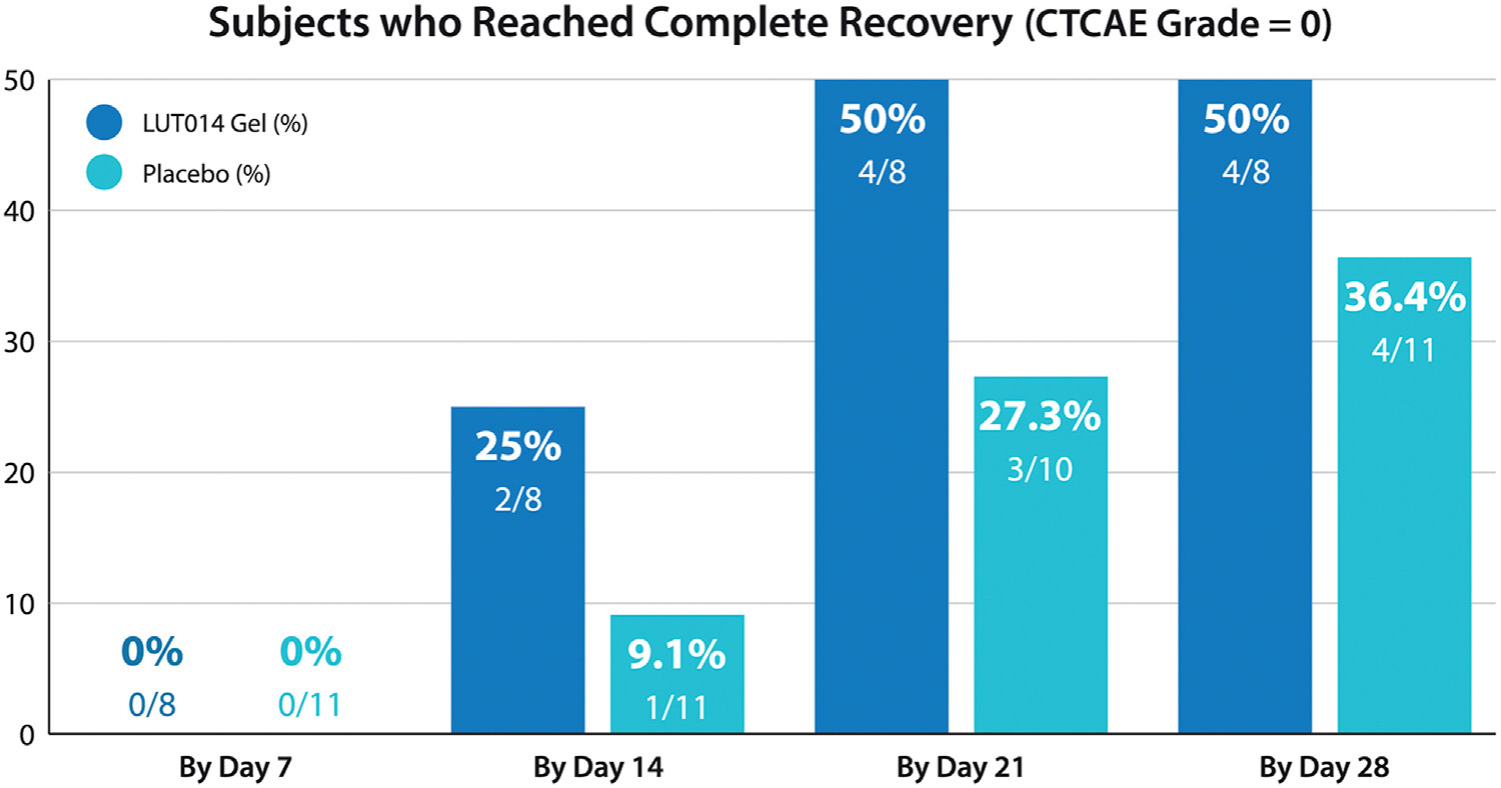
Rates of “complete recovery” as defined for the randomized component of the study. Note that none of the differences depicted reached statistical significance. *CTCAE*, Common terminology criteria for adverse events.

With regard to the primary end point – treatment success rate during the randomized portion of the study – all 8 women treated with LUT014 achieved treatment success (ie, had a reduction of at least 5 points in the Dermatology Life Quality Index score by day 14) in comparison to 73% of the placebo arm (8/11) at the same juncture (Fig 3). Furthermore, 75% of patients in the intervention arm showed improvement from grade 2 to grade 1 as early as day 7 in comparison to 55% of those assigned to the control arm. Moreover, 50% of the women assigned to the treatment arm experienced complete recovery at day 21 vs 27% of those receiving only placebo (*P* = .3765) (Fig 4). Overall, 4 out of 8 (50%) subjects had recovered completely in the LUT014 Gel group, while 4 out of 11 (36%) subjects had recovered completely in the placebo group by the end of the study (day 28) (*P* = .6577). None of these differences reached conventional levels of statistical significance. The mean and the median of the time to recovery were shorter in the LUT014 Gel than in the placebo group: 30.5 vs 46.6 days and 21.0 vs 54.0 days (*P* = .169), respectively.

## Discussion

We report findings of a phase I/II trial designed to assess a novel compound for the skin toxicity associated with irradiation of breast cancer. Such morbidity can lead to not only acute dermatitis but also chronic change.^23^

Our data suggest that LUT014 can be safely administered and that a positive signal may be associated with the compound. Although comparison between the 2 groups in the randomized portion of the trial did not reach conventional thresholds for statistical significance, it is noteworthy that considerably more women in the experimental arm had high body mass index when compared to those randomized to placebo. In an analysis of 1093 women, Behroozian et al^24^ found that high body mass index was a significant predictive factor related to radiation dermatitis. Whether high body mass index is a proxy for large breast size or, perhaps, reflective of the physiology of adipose tissue as an endocrine organ that releases cytokines which bring about more radiosensitivity is unknown.^25^

It is conceivable that more favorable results would have been observed among the subjects on the LUT014 intervention arm if the patients in the 2 arms had started to apply the gel at the same juncture relative to completion of RT. However, there was over a 1-day discrepancy with regards to initiating gel application. This shorter timeframe may have provided a “headstart” toward healing among patients receiving placebo. Ensuing phases of the clinical trials evaluating LUT014 will include methods to neutralize such a potential source of bias.

The pharmacological rationale for developing LUT014 for the treatment of RD is based on the role of the MAPK extracellular signal-regulated kinase 1/2 signaling pathway in inducing cell proliferation, the disruption of this process by radiation therapy, and the paradoxical activity of BRAF inhibitors in wild-type BRAF cells leading to activation rather than inhibition of the signaling pathway.^14^ Radiation therapy results in ionization events that damage DNA. Within the epidermis, this DNA damage disrupts normal proliferation and differentiation of basal keratinocytes, depleting the differentiated epidermal keratinocytes and ultimately resulting in loss of the protective barrier provided by the skin. We hypothesize that the paradoxical activity of a BRAF inhibitor could be leveraged in the setting of acute radiation dermatitis, where temporary hyperproliferation is desirable to repopulate the epidermal keratinocytes and restore the skin barrier.

We realize that there may be concerns raised since MAPK is part of the cancer development process. However, the topical application of LUT014 does not penetrate beyond the skin and does not lead to systemic exposure.^14^ Of note, we did not have any evidence of cancer lesion growth following irradiation and LUT014 application in our study. Accordingly, based on preclinical as well as clinical data, it is unlikely that radiation could permeate skin planes to reach tumor deposits at concentrations that would be capable of paradoxically activating the MAPK pathways in cancer cells.

A promising alternative approach for the management of radiation dermatitis is “prevention.” In a randomized trial, Behroozian et al^26^ compared a silicone-based polyurethane film (Mepitel) applied prophylactically to a standard intervention predicated on aqueous creams. The trial showed a significant reduction in grade 2 and grade 3 (common terminology criteria for adverse events v 5.0) skin reactions. While these results constitute thought-provoking level 1 evidence, the trial was comprised primarily of large-breasted women receiving conventional hypofractionation and was encumbered by several challenges including compromised adherence of the film from perspiration as well as patient perceptions that the film hindered daily activities. Another fascinating approach to prevention was offered by Kost et al^27^ who conducted a phase 2/3 trial based on bacterial decolonization with intranasal mupirocin ointment. Among 75 patients, none of the women receiving mupirocin developed grade 2 or higher RD vs 8 patients (22%) who developed ≥ grade 2 RD when treated on the control arm (*P* = .002). A limitation of that experience was the modification of the primary endpoint in the midst of the study. Indeed, when the prespecified primary end point was evaluated, there was only a trend toward beneficial effects which did not reach statistical significance. It is likely that prophylactic as well as therapeutic strategies will be needed in the management of RD.

Several limitations must be acknowledged. While promising results were detected, the ostensible benefits derived from LUT014 did not reach conventional levels of statistical significance. We believe that the results suggest a clinically meaningful benefit and plan to recruit greater numbers of subjects during the next phase of the trial. Another shortcoming of the results reported is the conduct of the trial at only 2 sites. Accordingly, the number of locations where the next phase of the study will be deployed will be expanded. Finally, we acknowledge the preponderance of white women enrolled on the study. This was unintentional; however, there is concern about generalizability of findings. In the next phase of study, methodologies will be invoked to be sure a more diverse group of subjects is offered enrollment.^28^

In conclusion we report promising but preliminary results from a novel compound for a clinical entity that compromises life quality. Our aim is to rigorously evaluate LUT014 by designing additional prospective trials assessing safety and efficacy.

## Conflicts of interest

Katz has consulted for and owns stock in Galera Therapeutics. Ciuba declares no potential conflict of interest. Ribas is a co-founder and member of the Board of Directors of Lutris Pharma. In addition, has received honoraria from consulting with CStone, Merck, and Vedanta, is or has been a member of the scientific advisory board and holds stock in Advaxis, Appia, Apricity, Arcus, Compugen, CytomX, Highlight, ImaginAb, ImmPact, ImmuneSensor, Inspirna, Isoplexis, Kite-Gilead, MapKure, Merus, PACT, Pluto, RAPT, Synthekine and Tango, has received research funding from Agilent and from Bristol-Myers Squibb through Stand Up to Cancer (SU2C), and patent royalties from Arsenal Bio. Shelach is an employee of Lutris Pharma and has written 5 families of patents pending and issued, regarding LUT014. Zellinger was employed by Lutris Pharma during the conduct of the trial and writing of the manuscript. Barrow declares no conflicts of interest. Corn works in a part-time capacity (0.2 FTE) as the chief medical officer of Lutris Pharma.
